# A New Method to Simultaneously Quantify the Antioxidants: Carotenes, Xanthophylls, and Vitamin A in Human Plasma

**DOI:** 10.1155/2016/9268531

**Published:** 2015-11-30

**Authors:** Mariel Colmán-Martínez, Miriam Martínez-Huélamo, Esther Miralles, Ramón Estruch, Rosa M. Lamuela-Raventós

**Affiliations:** ^1^Nutrition and Food Science Department, XaRTA, INSA, Pharmacy School, University of Barcelona, Avenue Joan XXII s/n, 08028 Barcelona, Spain; ^2^CIBER CB06/03 Fisiopatología de la Obesidad y la Nutrición, CIBEROBN, Choupana s/n, 15706 Santiago de Compostela, Spain; ^3^Scientific and Technical Services, University of Barcelona, Baldiri Reixac, 10, 08028 Barcelona, Spain; ^4^Department of Internal Medicine, Hospital Clínic, IDIBAPS, University of Barcelona, Villarroel, 170, 08036 Barcelona, Spain

## Abstract

A simple and accurate reversed phase high-performance liquid chromatography coupled with diode array detector (HPLC-DAD) method for simultaneously determining and quantifying the antioxidants carotenes, xanthophylls, and retinol in human plasma is presented in this paper. Compounds were extracted with hexane, a C30 column, and a mobile phase of methanol, methyl* tert*-butyl ether, and water were used for the separation of the compounds. A total of 8 carotenoids, 3* Z*-*β*-carotene isomers, and 1 fat-soluble vitamin (retinol) were resolved within 72 min at a flow rate of 0.6 mL/min. Detection was achieved at 450 nm for carotenoids and 330 nm for retinol. To evaluate the effectiveness of themethod, it has been applied to an intervention study conducted on eight volunteers.* Results*. Limits of detection were between 0.1 *μ*g/mL for lycopene and astaxanthin and 1.3 *μ*g/mL for 15-*Z*-*β*-carotene. Recoveries were ranged between 89% and 113% for *α*-carotene and astaxanthin, respectively. Accuracy was between 90.7% and 112.2% and precision was between 1% and 15% RSD. In human plasma samples compounds studied were identified besides three lycopene isomers, demonstrated to be suitable for application in dietary intervention studies.* Conclusions*. Due to its accuracy, precision, selectivity, and reproducibility, this method is suitable to dietary habits and/or antioxidants status studies.

## 1. Introduction

Several epidemiologic studies have shown that oxidative stress plays an essential role in the pathogenesis of many degenerative diseases, such as cancer, diabetes, age-related eye diseases, and cardiovascular diseases [1–6] and it has been suggested that antioxidants may exert a protective role against these chronic diseases by defending against oxidative damage [7, 8]. There is an increasing interest in the analysis of carotenoids and some fat-soluble vitamins such as retinol (vitamin A) due to their antioxidant properties and their relationship with the prevention of chronic diseases [9–12]. The characterization and quantification of carotenoids and retinol in human plasma are essential for best interpretation of epidemiologic studies linking oxidative stress, diet, and health.

Carotenoids comprise a group of fat soluble phytochemicals widely distributed in nature, responsible for the colours of many fruits and vegetables, as well as certain animal tissues and leaf coloration after the degradation of chlorophyll [13]. Considering the number of double bonds in the molecules, carotenoids can be found with* cis* or* trans* configuration (or* E/Z* isomers). In general the all-*E* form is predominant in nature but numerous researches show that more than 50% of some carotenoids, as lycopene, present in human plasma and tissues are* Z*-isomers [14] and it is believed that geometrical configuration of carotenoids could have implications in the solubility, absorption, and transport in humans [15, 16] or even geometrical isomers of provitamin A carotenoids have different vitamin A activities [17]. The enhanced absorption of lycopene* Z*-isomers is hypothesised to result from higher solubility in mixed micelles, the shorter length of the* Z*-isomers, and/or a lower tendency to aggregate [14, 18]. This hypothesis was supported by studies in both animals and humans [18, 19].

Among fat-soluble vitamins, retinol exerts an important antioxidant action via the inhibition of lipid peroxidation and has free-radical-scavenging properties [11, 20]; meanwhile, carotenoids are known to be effective quenchers of singlet oxygen, as well as strong scavengers of different reactive oxygen species (ROS) [21].

High-performance liquid chromatography (HPLC) is one of the most used techniques for the identification of carotenoids [22, 23] as well as vitamin A in human plasma [24–26]. The advantages of the use of this technique are speed, sensitivity, and accuracy for the determination of the compounds in addition to the economy of the solvents required and the simple coupling with other techniques.

In light of the importance of these compounds for health maintenance, an accurate determination and quantification in plasma is necessary. The aim of this study is to develop a simple HPLC method to identify the main carotenoids and their geometrical isomers as well as retinol, one of the major antioxidant fat-soluble vitamins, in human plasma.

## 2. Experimental Procedures

### 2.1. Materials and Methods

#### 2.1.1. Reagents and Standards

Carotenoids and vitamins standards: retinol, astaxanthin, lutein, zeaxanthin,* E*-*β*-apo-8′-carotenal, cryptoxanthin, 15-*Z*-*β*-carotene, 13-*Z*-*β*-carotene, *α*-carotene, *β*-carotene, 9-*Z*-*β*-carotene, and lycopene were purchased from Sigma-Aldrich (St. Louis, MO, USA).

Methanol (MeOH) and methyl-*tert-*butyl ether (MTBE) of HPLC grade were obtained from Panreac Quimica SA (Barcelona, Spain). Ultrapure water (Milli-Q) was generated by a Millipore System (Bedford, MA, USA). Human plasma and butylated hydroxytoluene (BHT) were acquired from Sigma-Aldrich.

#### 2.1.2. Preparation of Standard and Stock Solutions

Individual working standards of retinol, astaxanthin, lutein, zeaxanthin,* E*-*β*-apo-8′-carotenal, cryptoxanthin, 15-*Z*-*β*-carotene, 13-*Z*-*β*-carotene, *α*-carotene, *β*-carotene, 9-*Z*-*β*-carotene, and lycopene were prepared at a concentration of 1 mg/mL in MTBE. All working standards were manipulated under protection of light to minimise light-induced isomerisation, stored in eppendorf tubes, and kept at −20°C until use.

The stock solution used to spike plasma samples was prepared by mixing individual working standards at a concentration of 50 mg/mL in MTBE.

#### 2.1.3. Extraction and Isolation of Carotenoids and Retinol

Plasma was subjected to a liquid-liquid extraction procedure previously described by our working group [27]. Briefly, 800 *μ*L of plasma was mixed with 800 *μ*L of ethanol and 2 mL of hexane/BHT (100 mg/L). Then, 300 *μ*L of stock solution was added followed by a vortex-mixing for 1 minute and centrifuged at 2062 gr for 5 minutes at 4°C. The upper nonpolar layer was removed and the remaining aqueous plasma mixture was reextracted as described above. The two nonpolar extracts were combined in a glass vial and dried under nitrogen gas at <25°C followed by a reconstitution with 300 *μ*L of MTBE. Then, the samples were stored into insert-amber vials for HPLC at −20°C until the day of analysis.

#### 2.1.4. Instrumentation

Chromatographic analysis was carried out in an HP 1100 HPLC system (Hewlett-Packard, Waldbronn, Germany), consisting of a quaternary pump and an autosampler coupled to a diode array detector DAD G1315B. Chromatographic separation was performed on a reversed-phase column YMC Carotenoid S-5 *μ*m, 250 mm × 4.6 mm (Waters, Milford, MA), maintained at 25°C, and connected to a precolumn YMC Guard Cartridge Carotenoid 20 × 4.0 mm i.d, S-5 *μ*m. The guard column was replaced every 500 injections. The integration was performed with Agilent ChemStation Software. The DAD detector was adjusted at 450 nm for the carotenoids and 330 nm for retinol, respectively. All compounds were identified by retention time compared with pure standards as well as by the UV-Vis spectra of each compound.

#### 2.1.5. Chromatographic Conditions

The chromatographic separation was performed using the following solvents: Milli-Q water (A), methanol (B), and MTBE (C). Solvent A was used isocratically at 4% while the following linear gradient was used for B (*t* (min), %B): (0.0, 90); (40.0, 40); (60.0, 6); (62.0, 90); (72.0, 90). Twenty microliter aliquots of the samples were injected in the HPLC-DAD system. Total run time was 72 minutes at a flow rate of 0.6 mL/min.

#### 2.1.6. Quality Parameters

The method was fully validated based on the criteria of the AOAC International and the U.S. Department of Health and Human Services Food and Drug Administration (FDA) [28, 29]. The quality parameters established for the validation of the method were accuracy, intra- and interday precision, recovery, limit of detection (LoD), limit of quantification (LoQ), and linearity.

Three dilutions were prepared from the stock solution in MTBE to the final concentrations of low (1.5 *μ*g/mL), medium (4 *μ*g/mL), and high (9 *μ*g/mL) for all analytes for precision and accuracy assays.

Accuracy consisted in the closeness of agreement between the measured value and the reference value and was established by repetitively spiking blank plasma with three known concentrations of analyte standards: low, medium, and high with respect to the calibration curves, in five replicates. The results were determined as the percentage of the ratio of the mean observed concentration and the known spiked concentration in the biological matrices. The mean value should be within ±15% of the nominal value.

Intraday precision and interday precision were considered using five determinations per three concentration levels: low, medium, and high in a single analytical run or in three different days, respectively. The precision of the method was assessed on the % RSD (percentage of relative standard deviation) of intra- and interday repeatability and the values determined at each concentration level should not exceed 15% of RSD, according to the regulation of the AOAC and FDA.

Recovery was accomplished by preparing seven-point calibration curves and seven-point external curves, spiked before and after extraction, respectively. The detector response obtained from the amount of analyte added to and extracted from the biological matrix was compared to the detector response obtained for the same concentration of the pure authentic standard. A linear regression between the ratio analyte concentration against the calculated concentration has been applied, and the slope multiplied by 100 corresponded to the analyte recovery.

Limit of detection (LoD) and limit of quantification (LoQ) were determined by comparing measured signals from samples with the low concentration of analyte with those of blank samples and establishing the minimum concentration at which the analyte can be reliably detected or quantified. A signal-to-noise ratio in the order of 3 : 1 and 10 : 1, for LoD and LoQ, respectively, was considered acceptable.

Linearity was tested by assessing signal responses of target analytes from plasma samples spiked in duplicate at seven different concentrations and by calculation of linear regression.

### 2.2. Method Application: Pilot Dietary Intervention Study

#### 2.2.1. Biological Material

To assess the efficiency in the identification of carotenoids and retinol in human plasma, the present method was applied to a small-scale prospective, open and controlled, single-arm intervention study conducted in eight volunteers free of cardiovascular disease but with high risk of developing it, aged 69.9 ± 3.8 years with a mean body mass index of 32.3 ± 3.8 kg/m^2^.

The volunteers were instructed to avoid the consumption of tomato and tomato-based products 3 days before the study. On the experimental day, after 12 hours of fasting, blood samples were collected early in the morning to quantify carotenoids and retinol as baseline. After that, all volunteers have followed a similar diet developed by a trained dietitian, which took into account their preferences and tastes, as well as the consideration that participants were diabetic, obese, hypertensive, and/or dyslipidemic. Each day during 4 weeks, participants consumed 250 mL of tomato juice before dinner and at the end of the study blood samples were taken for comparing with baseline. All samples were stored at −80°C until analysis.

The study protocol was approved by the Ethics Committee of Clinical Investigation of the University of Barcelona (Spain), and the clinical trial was registered at the International Standard Randomized Controlled Trial Number (ISRCTN20409295). Written informed consent was obtained from all participants.

### 2.3. Statistical Analysis

Statistical analysis was performed using the SPSS Statistical Analysis System (version 22.0; SPSS Inc, Chicago, IL). Data are presented as means and standard deviation (SD). Statistical differences between the two interventions were analysed by the nonparametric statistical Wilcoxon test for paired comparisons. Statistical significance was set at *p* < 0.05.

## 3. Results and Discussion

### 3.1. HPLC Method Development

#### 3.1.1. Extraction

For the extraction of carotenoids and retinol, precipitation of proteins from plasma as a prior step is required. To achieve this issue, most methods use organic solvents such as methanol, ethanol, or acetonitrile [30]. Some studies use ethanol-BHT at different concentrations [31, 32] while others use ethanol-ascorbic acid [33] or ethanol-saline [34] for protection of carotenoids besides deproteinization. Different solvents and solvent combinations have been described in the literature for removing lipophilic analytes from biosamples. Hexane alone [35, 36] or combined with other solvents, such as hexane/acetone [37], hexane/ether [34], or hexane/ethanol/acetone/toluene [38], seems to be the most used solvents for the extraction of carotenoids and lipophilic compounds from plasma. There are few studies where hexane-BHT is used for protection of carotenoids during the extraction [39, 40] while other authors prefer to use hexane-saline [34]. Both mixtures, hexane-BHT and hexane-saline, were tested for extraction. Recoveries obtained with hexane-saline were between 57.4% and 86.9% corresponding to* E*-lycopene and 13-*Z*-*β*-carotene, respectively, versus recoveries range between 73.4% and 90.4% corresponding to* E*-lycopene and *β*-carotene, respectively, using hexane-BHT as extraction solvent. Due to its simplicity, speed, and good recoveries achieved, ethanol for deproteinization and repeated extraction with hexane-BHT were chosen for performing this study.

#### 3.1.2. Separation and Identification

Numerous HPLC methods are reported for separation and identification of carotenoids and fat-soluble vitamins from diverse complex matrices such as food, food products, or plasma. The use of isocratic or gradient systems coupled to different types of columns and/or coupled to different detectors or even the use of different temperatures for the stability of the analytes, depending of the compounds studied is the best described technique for these purposes.

**Figure 1:**
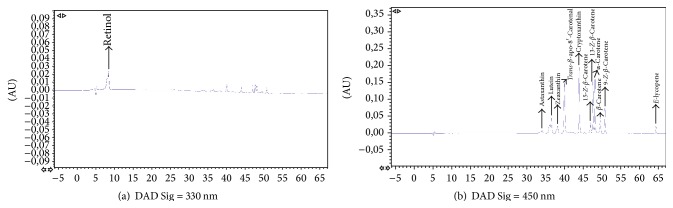
Representative HPLC chromatogram of carotenoids and retinol standards in MTBE.

**Figure 2:**
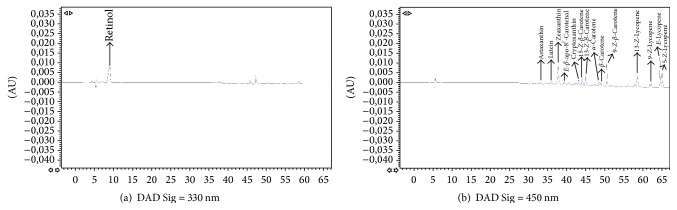
Representative HPLC chromatogram of carotenoids and retinol in human plasma corresponding to a volunteer after tomato juice intervention.

Reversed-phase HPLC with C_18_ columns and isocratic or gradient elution seems to be the modality most commonly used for identification and quantification of carotenoids and fat-soluble vitamins [41–44]. One of the major problems in the identification of carotenoids and lipophilic compounds using C_18_ columns seems to be the separation of geometrical isomers of carotenoids [41–47]. Tzeng et al. [33] have developed an isocratic method for the identification of carotenoids using a C_18_ column, but they could only separate three carotenoids: lutein, lycopene, and *β*-carotene. Olmedilla et al. [40] also have described a gradient method with a C_18_ column but could not identify isomers of the carotenoids. To solve this issue, polymeric C_30_ columns were developed for separation of* Z-E* isomers [22, 48, 49]. The use of this kind of column has allowed us the separation of the twelve compounds studied, including 3* Z*-isomers of *β*-carotene and another 3* Z*-isomers of lycopene. Figures 1 and 2 show a representative HPLC-DAD chromatogram of carotenoids and retinol in a standard mixture and a human plasma sample, respectively.

Likewise lutein and zeaxanthin are reported as two difficult compounds to separate on monomeric C_18_ columns [42, 43]. Gueguen et al. [44] found an inadequate resolution of lutein and zeaxanthin with the method proposed using a C_18_ column and an isocratic elution system; Thibeault et al. [31] were not able to differentiate lutein and zeaxanthin with these same conditions. A high resolution can be achieved by using polymeric C_30_ columns with gradient elution [22, 49, 50]. With the present method, all analytes, including lutein and zeaxanthin, have reached a resolution higher than 1.5, indicating a good separation of the compounds and a good symmetry of all peaks. Results are shown in Table 1.

Coupling of photodiode array detector (DAD) and fluorescence detector (FLD) is the technique chosen by Epler et al. [51] and Liu et al. [32] for identification of carotenoids and retinols from human serum and foods. Using more complex techniques, such as photoisomerization of some analytes as a prior step, Ferruzzi et al. [49] identified 13 lycopene isomers (*Z*/*E*) by using an electrochemical detector (ECD). Lyan et al. [46] and Lee et al. [47, 52] have identified various carotenoids by the coupling of two different detectors or columns. Other authors like Gleize et al. [53] have set up a gradient method for the identification of eleven carotenoids and fat-soluble vitamins from complex matrixes such as food samples, human plasma, and human adipose tissue using a single C_30_ column kept at 35°C. All these techniques allow the separation and identification of lipid compounds such as carotenoids and fat soluble vitamins from different matrices but are complex and require coupling of columns and use of temperature or combination of detectors, which is not always possible in all laboratories.

In this study, carotenoids and retinol were separated in a single run on a reversed-phase column using a gradient system of water, methanol, and MTBE. The mobile phase was optimized in order to obtain the best separation of the compounds in the shortest time possible and to achieve this, several gradients were assessed. The best results obtained for the conditions were described in the chromatographic conditions section.

### 3.2. Validation Parameters

#### 3.2.1. Linearity

According to the maximal reported value in plasma for each analyte, plasma samples were spiked in duplicate at seven different concentrations ranged from LoQ of each compound to 10 *μ*g/mL. The analytical procedure was linear over the concentration range tested with the correlation coefficient from 0.9952 to 0.9984 for all compounds in plasma samples, demonstrating a good linearity of the curves.  Table 4 summarizes the correlation coefficients of the curves of all compounds.

#### 3.2.2. Accuracy and Precision

Accuracy and intra and interday precision were studied. All compounds analysed met the acceptance criteria to not overcome 15% RSD in both intra- and interday precision and in the three concentration levels. The highest values were 15% belonging to astaxanthin, zeaxanthin, *α*-carotene, *β*-carotene, and 13-*Z*-*β*-carotene. Accuracy results obtained were between 90.7% and 112.2% being within limits of accuracy, 85–115%. The method proposed demonstrated good accuracy and precision in plasma samples, asserting that was feasible for the determination of carotenoids and retinol in human plasma. Results are expressed in Tables 2 and 3.

#### 3.2.3. Recovery

Recovery for retinol was 96%, similar to the value achieved by Kanďár et al. [54]. For carotenoids, the recoveries were between 89% and 113%, corresponding to *α*-carotene and astaxanthin, respectively. Comparing our results with those reported by Talwar et al. [43] we achieved a recovery 18% higher for lutein and 9% higher for *β*-carotene with the described method; comparing with the data presented by Tzeng et al. [33], we achieved a better recovery for lutein (20% higher), and comparing with Rajendran et al. [22] we obtained a better recovery for lutein (19% higher), zeaxanthin (13% higher), and cryptoxanthin (7% higher). Karppi et al. [39] have reported similar values of recovery except for lutein, zeaxanthin, and *β*-carotene that were 10%, 17%, and 16%, respectively, lower than in the present study.

The extraction procedure was really effective, since high recoveries can be observed in Table 2.

#### 3.2.4. Limit of Detection (LoD) and Limit of Quantification (LoQ)

The LoD found was 0.1 *μ*g/mL for astaxanthin and lycopene; 0.2 *μ*g/mL for retinol, zeaxanthin,* E*-*β*-apo-8′-carotenal, cryptoxanthin, *β*-carotene, and 9-*Z*-*β*-carotene; 0.4 *μ*g/mL for lutein and 13-*Z*-*β*-carotene; 0.5 *μ*g/mL for *α*-carotene, and 1.3 *μ*g/mL for 15-*Z*-*β*-carotene. The LoQ was from 0.3 *μ*g/mL to 4.4 *μ*g/mL for astaxanthin and 15-*Z-β*-carotene, respectively. All LoD and LoQ values obtained are expressed in Table 4. In a study carried out by Mitrowska et al. [55] similar values were obtained for* E*-*β*-apo-8′-carotenal, astaxanthin, and lycopene. In another study developed by Talwar et al. [43] a similar value for retinol but not for the other compounds studied can be observed; nonetheless, with the presented method we have achieved the separation and identification of 11 carotenoids and 1 fat-soluble vitamin in a single run.

#### 3.2.5. Plasma Levels of Carotenoids and Retinol

The method was used for measuring concentrations of carotenoids and retinol in human plasma samples. All compounds were determined following the procedure described above and the results are expressed in Table 5.

Nine of the 12 validated analytes were identified in plasma at baseline: retinol, astaxanthin, lutein,* E*-*β*-apo-8′-carotenal, cryptoxanthin, 13-*Z-β*-carotene, *α-*carotene, *β-*carotene, and lycopene. It is important to highlight that, besides the identification of the validated compounds, we also have searched for* cis* isomers of lycopene which have been suggested to be more bioavailable than* E*-lycopene, typically found in raw food [18]. In fact, in human plasma, total lycopene is an isomeric mixture containing 40% to 50% as* Z-*isomers [5] and according to the antioxidant properties of lycopene, from greatest to least are 5-*Z*, 9-*Z*, 7-*Z*, 13-*Z*, 15-*Z*, 11-*Z,* and all-*E* lycopene [56]. We have identified 5-*Z*, 9-*Z,* and 13-*Z* lycopene, all present in human plasma according to the study carried out by Arranz et al. [27]

After tomato juice intervention, an increase in retinol, astaxanthin, cryptoxanthin, 13-*Z-β-*carotene, *β-*carotene, 13-*Z*-lycopene, 9-*Z*-lycopene,* E*-lycopene, and 5-*Z*-lycopene was observed, with values between 5.194 and 0.140 *μ*g/mL corresponding to* E*-lycopene and 13-*Z-β-*carotene, respectively. Among these compounds, lycopene and its isomers have presented a significant increase after tomato juice consumption (*p* < 0.05). Results are shown in Table 5.

In a study carried out by Pellegrini et al. [57], they found a concentration of 0.31 *μ*g/mL of lycopene and 0.17 *μ*g/mL of *β*-carotene in human plasma after a consumption of tomato purée. In another study performed by Porrini et al. [58], they have seen values in the order of 0.18, 0.21, 0.02, 0.13, 0.03, and 0.23 *μ*g/mL for lycopene, lutein, zeaxanthin, *β*-cryptoxanthin, *α*-carotene, and *β*-carotene, respectively, after a daily supplementation of tomato purée. Gärtner et al. [59] have determined values of 0.02, 0.0002, and 0.001 *μ*g/mL for lycopene, *α*-carotene, and *β*-carotene, respectively, after a consumption of tomato paste. Regarding retinol, the results obtained with the present method are higher than those reported by Liu et al. [32] who have found a mean of 0.55 *μ*g/mL and 0.56 *μ*g/mL in plasma of women and men, respectively, participants of the Toronto Nutrigenomics and Health Study.

## 4. Conclusions

Based on previous reported work, the present method is simple, accurate, reliable, sensitive, and selective for the determination of the antioxidants carotenoids and retinol in human plasma samples. With this method, all analytes of interest were successfully resolved, including lutein, zeaxanthin, and* Z*-isomers of *β*-carotene which have previously been reported as critical compounds to identify and separate. The sample preparation procedure in this method provides excellent recoveries for all analytes.

A total of 8 carotenoids, 3* Z*-isomers of the *β*-carotene, 1 fat-soluble vitamin, and also 3* Z*-isomers of the lycopene were simultaneously separated and identified in human plasma by the use of polymeric C_30_ chromatography column with a gradient elution.

Future approaches to enhance the analysis of the isomers should focus on the conclusive identification of the compounds that remain tentatively identified. For this purpose, the isolation of standards of most* cis*-isomers and the determination of their absorption coefficients are needed for their accurate quantification.

The HPLC method was completely validated and due to the good results obtained after the intake of tomato juice, this method may be applied to evaluate the liposoluble antioxidants carotenoids and vitamin A in clinical intervention antioxidants trials and epidemiological studies status investigations.

## Figures and Tables

**Table 1:** Resolution of the analytes studied.

Analyte	Rt (min)	Wavelength (nm)	Width (min)	Resolution
Retinol	8.54	330	0.6	N.d.
Astaxanthin	34.07	450	0.38	2.3
Lutein	36.55	450	0.72	1.4
Zeaxanthin	38.24	450	0.53	2.2
*E-β-*apo-8′-carotenal	40.01	450	0.29	7.7
Cryptoxanthin	43.94	450	0.22	6.9
15-*Z-β*-carotene	46.96	450	0.22	1.4
13-*Z-β*-carotene	47.57	450	0.21	1.3
*α*-carotene	48.12	450	0.2	2.9
*β*-carotene	49.57	450	0.31	2.3
9-*Z-β*-carotene	50.76	450	0.2	33.0
*E*-lycopene	64.29	450	0.21	33.0

N.d.: not determined.

*R* = 2[(Rt)*B*  − (Rt)*A*]/(*WA* + *WB*).

*R* = resolution.

Rt = retention time.

*W* = width.

**Table 2:** Accuracy and recovery of the compounds studied.

Analyte	Accuracy (%)	Recoveries (%)
Retinol	105 ± 9	96 ± 3
Astaxanthin	99 ± 7	113 ± 6
Lutein	99 ± 13	112 ± 9
Zeaxanthin	101 ± 11	107 ± 5
*E-β-*apo-8′-carotenal	98 ± 10	94 ± 3
Cryptoxanthin	103 ± 12	96 ± 3
15-*Z-β*-carotene	90 ± 13	101 ± 2
13-*Z*-*β*-carotene	105 ± 12	92 ± 5
*α*-carotene	98 ± 12	89 ± 4
*β*-carotene	97 ± 13	96 ± 2
9-*Z*-*β*-carotene	112 ± 7	93 ± 3
*E*-lycopene	100 ± 12	91 ± 3

**Table 3:** Intra- and interday precision.

Analyte	Precision											
	1,5 *µ*g/mL (*n* = 5)				6 *µ*g/mL (*n* = 5)				9 *µ*g/mL (*n* = 5)			
	Day 1 (RSD %)	Day 2 (RSD %)	Day 3 (RSD %)	RSD Interday	Day 1 (RSD %)	Day 2 (RSD %)	Day 3 (RSD %)	RSD Interday	Day 1 (RSD %)	Day 2 (RSD %)	Day 3 (RSD %)	RSD Interday
Retinol	3	4	5	4.9	10	12	8	14.1	13	10	11	14.3
Astaxanthin	7	3	3	14.5	11	13	12	14.6	15	12	12	14.7
Lutein	8	9	4	14.2	12	14	8	14.3	10	9	12	13.3
Zeaxanthin	4	15	4	10.8	12	3	7	12.9	11	11	10	14.8
*E*-*β*-apo-8′-carotenal	11	10	4	14.3	11	12	8	14.7	13	9	12	13.8
Cryptoxanthin	5	13	3	12.1	11	6	9	14.3	11	10	8	13.9
15-*Z*-*β*-carotene	5	13	1	9.4	11	5	9	15.1	10	7	11	13.4
13-*Z*-*β*-carotene	6	14	3	14.9	12	15	12	14.0	11	10	10	14.9
*α*-carotene	4	15	2	9.4	11	15	11	13.7	11	9	10	14.8
*β*-carotene	11	10	8	11.2	11	15	14	14.9	12	6	10	12.8
9-*Z*-*β*-carotene	6	10	7	10.4	12	12	4	13.9	11	11	11	14.8
*E*-lycopene	5	5	2	11.6	10	13	9	13.8	10	15	8	14.8

N.d.: not determined.

RSD: relative standard deviation.

**Table 4:** Limit of detection (LoD), limit of quantification (LoQ), range of concentration, calibration curve, and correlation coefficient of the analytes in blank plasma spiked with standard solution.

Analytes	LoD (*µ*g/mL)	LoQ (*µ*g/mL)	Linearity range (*µ*g/mL)	Calibration curve	Correlation coefficient (*r*)
Retinol	0.2	0.7	0.7–10	*y* = 69.684*x* − 15.857	0.9952
Astaxanthin	0.1	0.3	0.3–10	*y* = 49.138*x* + 3.0197	0.9964
Lutein	0.4	1.3	1.3–10	*y* = 119.37*x* − 2.263	0.9954
Zeaxanthin	0.2	0.7	0.7–10	*y* = 69.14*x* + 1.53	0.9955
Apo-8′-carotenal	0.2	0.7	0.7–10	*y* = 249.4*x* − 20.908	0.9957
Cryptoxanthin	0.2	0.7	0.7–10	*y* = 200.48*x* − 15.759	0.9952
15-Z-*β*-carotene	1.3	4.3	4.3–10	*y* = 91.235*x* − 10.848	0.9984
13-Z-*β*-carotene	0.4	1.3	1.3–10	*y* = 90.591*x* + 8.7376	0.9969
*α*-carotene	0.5	1.6	1.6–10	*y* = 243.07*x* − 37.135	0.9966
*β*-carotene	0.2	0.7	0.7–10	*y* = 105.8*x* − 2.9437	0.9983
9-Z-*β*-carotene	0.2	0.7	0.7–10	*y* = 75.685*x* − 8.4189	0.9959
*E*-lycopene	0.1	0.3	0.3–10	*y* = 34.532*x* − 4.2861	0.9952

**Table 5:** Carotenoids and retinol in human plasma before (baseline) and after the dietary intervention.

Analytes	Concentration			
	Baseline	After intervention	Baseline	After intervention
	Mean ± SD (*µ*g/mL)	Mean ± SD (*µ*g/mL)	Mean ± SD (*µ*mol/L)	Mean ± SD (*µ*mol/L)
Retinol	1.82 ± 0.37	1.90 ± 0.49	3.46 ± 0.70	3.62 ± 0.93
Astaxanthin	0.76 ± 0.44	0.88 ± 0.37	1.27 ± 0.74	1.48 ± 0.63
Lutein	0.07 ± 0.02	0.06 ± 0.06	0.13 ± 0.03	0.11 ± 0.11
Zeaxanthin	N.d.	N.d.	N.d.	N.d.
*E-β*-apo-8′-carotenal	0.57 ± 0.03	0.57 ± 0.03	1.38 ± 0.06	1.38 ± 0.08
Cryptoxanthin	0.18 ± 0.12	0.20 ± 0.07	0.32 ± 0.22	0.37 ± 0.12
15-*Z*-*β*-carotene	N.d.	N.d.	N.d.	N.d.
13-*Z*-*β*-carotene	0.13 ± 0.00	0.14 ± 0.02	0.23 ± 0.00	0.26 ± 0.04
*α*-carotene	0.22 ± 0.00	N.d.	0.41 ± 0.00	N.d.
*β*-carotene	0.95 ± 0.50	1.09 ± 0.53	1.77 ± 0.93	2.03 ± 0.98
9-Z-*β*-carotene	N.d.	N.d.	N.d.	N.d.
13-*Z*-lycopene	N.d.^a^	2.79 ± 1.44^a^	N.d.^a^	5.20 ± 2.69^a^
9-Z-lycopene	N.d.^a^	0.38 ± 1.42^a^	N.d.^a^	0.71 ± 2.64^a^
*E*-lycopene	1.15 ± 0.83^a^	5.19 ± 2.35^a^	2.14 ± 1.54^a^	9.67 ± 4.38^a^
5-*Z*-lycopene	0.75 ± 1.10^a^	3.07 ± 1.43^a^	1.41 ± 2.06^a^	5.72 ± 2.67^a^

N.d.: not determined.

SD: standard deviation.

^a^Values in a row with the same letter are significantly different (*p* < 0.05). Data analyzed by Wilcoxon test for repeated measures.

## References

[1] Valko M., Rhodes C. J., Moncol J., Izakovic M., Mazur M. (2006). Free radicals, metals and antioxidants in oxidative stress-induced cancer. *Chemico-Biological Interactions*.

[2] Vokurkova M., Xu S., Touyz R. M. (2007). Reactive oxygen species, cell growth, cell cycle progression and vascular remodeling in hypertension. *Future Cardiology*.

[3] Halliwell B. (2005). Free Radicals and other reactive species in Disease. *eLS*.

[4] Tanaka T., Shnimizu M., Moriwaki H. (2012). Cancer chemoprevention by carotenoids. *Molecules*.

[5] Dillingham B., Rao A. V. (2009). Biologically active lycopene in human health. *International Journal of Naturopathic Medicine (San Francisco)*.

[6] Jeurnink S. M., Ros M. M., Leenders M. (2015). Plasma carotenoids, vitamin C, retinol and tocopherols levels and pancreatic cancer risk within the European prospective investigation into cancer and nutrition: a nested case-control study: plasma micronutrients and pancreatic cancer risk. *International Journal of Cancer*.

[7] McCall M. R., Frei B. (1999). Can antioxidant vitamins materially reduce oxidative damage in humans?. *Free Radical Biology and Medicine*.

[8] Tapiero H., Townsend D. M., Tew K. D. (2004). The role of carotenoids in the prevention of human pathologies. *Biomedicine and Pharmacotherapy*.

[9] Krinsky N. I., Johnson E. J. (2005). Carotenoid actions and their relation to health and disease. *Molecular Aspects of Medicine*.

[10] Ciccone M. M., Cortese F., Gesualdo M. (2013). Dietary intake of carotenoids and their antioxidant and anti-inflammatory effects in cardiovascular care. *Mediators of Inflammation*.

[11] Yuan J.-M., Gao Y.-T., Ong C.-N., Ross R. K., Yu M. C. (2006). Prediagnostic level of serum retinol in relation to reduced risk of hepatocellular carcinoma. *Journal of the National Cancer Institute*.

[12] Gey K. F., Ducimetière P., Evans A. (2010). Low plasma retinol predicts coronary events in healthy middle-aged men: the PRIME Study. *Atherosclerosis*.

[13] Stahl W., Sies H. (2005). Bioactivity and protective effects of natural carotenoids. *Biochimica et Biophysica Acta—Molecular Basis of Disease*.

[14] Boileau T. W., Boileau A. C., Erdman J. W. (2002). Bioavailability of all-trans and cis-isomers of lycopene. *Experimental Biology and Medicine*.

[15] Britton G. (1995). Structure and properties of carotenoids in relation to function. *The FASEB Journal*.

[16] Schieber A., Carle R. (2005). Occurrence of carotenoid cis-isomers in food: technological, analytical, and nutritional implications. *Trends in Food Science and Technology*.

[17] Scott K. J., Rodriquez-Amaya D. (2000). Pro-vitamin A carotenoid conversion factors: retinol equivalents—fact or fiction?. *Food Chemistry*.

[18] Stahl W., Sies H. (1992). Uptake of lycopene and its geometrical isomers is greater from heat-processed than from unprocessed tomato juice in humans. *Journal of Nutrition*.

[19] Boileau A. C., Merchen N. R., Wasson K., Atkinson C. A., Erdman J. W. (1999). Cis-lycopene is more bioavailable than trans-lycopene in vitro and in vivo in lymph-cannulated ferrets. *Journal of Nutrition*.

[20] Rózanowska M., Cantrell A., Edge R., Land E. J., Sarna T., Truscott T. G. (2005). Pulse radiolysis study of the interaction of retinoids with peroxyl radicals. *Free Radical Biology and Medicine*.

[21] Mortensen A., Skibsted L. H., Truscott T. G. (2001). The interaction of dietary carotenoids with radical species. *Archives of Biochemistry and Biophysics*.

[22] Rajendran V., Pu Y. S., Chen B. H. (2005). An improved HPLC method for determination of carotenoids in human serum. *Journal of Chromatography B: Analytical Technologies in the Biomedical and Life Sciences*.

[23] Nakagawa K., Kiko T., Hatade K. (2008). Development of a high-performance liquid chromatography-based assay for carotenoids in human red blood cells: application to clinical studies. *Analytical Biochemistry*.

[24] Casal S., Macedo B., Oliveira M. B. P. P. (2001). Simultaneous determination of retinol, *β*-carotene and *α*-tocopherol in adipose tissue by high-performance liquid chromatography. *Journal of Chromatography B: Biomedical Sciences and Applications*.

[25] Andreoli R., Manini P., Poli D., Bergamaschi E., Mutti A., Niessen W. M. (2004). Development of a simplified method for the simultaneous determination of retinol, *α*-tocopherol, and *β*-carotene in serum by liquid chromatography-tandem mass spectrometry with atmospheric pressure chemical ionization. *Analytical and Bioanalytical Chemistry*.

[26] Ortega H., Coperías J. L., Castilla P., Gómez-Coronado D., Lasunción M. A. (2004). Liquid chromatographic method for the simultaneous determination of different lipid-soluble antioxidants in human plasma and low-density lipoproteins. *Journal of Chromatography B: Analytical Technologies in the Biomedical and Life Sciences*.

[27] Arranz S., Martínez-Huélamo M., Vallverdu-Queralt A. (2015). Influence of olive oil on carotenoid absorption from tomato juice and effects on postprandial lipemia. *Food Chemistry*.

[28] Lynch J., Editor C. Appendix E: Laboratory Quality Assurance.

[29] Food and Drug Administration (2013). *Draft Guidance for Industry Bioanalytical Method Validation*.

[30] Bruce S. J., Tavazzi I., Parisod V., Rezzi S., Kochhar S., Guy P. A. (2009). Investigation of human blood plasma sample preparation for performing metabolomics using ultrahigh performance liquid chromatography/mass spectrometry. *Analytical Chemistry*.

[31] Thibeault D., Su H., MacNamara E., Schipper H. M. (2009). Isocratic rapid liquid chromatographic method for simultaneous determination of carotenoids, retinol, and tocopherols in human serum. *Journal of Chromatography B: Analytical Technologies in the Biomedical and Life Sciences*.

[32] Liu Z., Lee H.-J., Garofalo F., Jenkins D. J. A., El-Sohemy A. (2011). Simultaneous measurement of three tocopherols, all-trans-retinol, and eight carotenoids in human plasma by isocratic liquid chromatography. *Journal of Chromatographic Science*.

[33] Tzeng M., Yang F., Chen B. (2004). Determination of major carotenoids in human serum by liquid chromatography. *Journal of Food and Drug Analysis*.

[34] Melendez-Martinez A. J., Stinco C. M., Liu C., Wang X.-D. (2013). A simple HPLC method for the comprehensive analysis of cis/trans (Z/E) geometrical isomers of carotenoids for nutritional studies. *Food Chemistry*.

[35] Nomura A. M., Stemmermann G. N., Lee J., Craft N. E. (1997). Serum micronutrients and prostate cancer in Japanese Americans in Hawaii. *Cancer Epidemiology, Biomarkers ’ Prevention*.

[36] Su Q., Rowley K. G., Balazs N. D. H. (2002). Carotenoids: separation methods applicable to biological samples. *Journal of Chromatography B: Analytical Technologies in the Biomedical and Life Sciences*.

[37] Ferruzzi M. G., Nguyen M. L., Sander L. C., Rock C. L., Schwartz S. J. (2001). Analysis of lycopene geometrical isomers in biological microsamples by liquid chromatography with coulometric array detection. *Journal of Chromatography B: Biomedical Sciences and Applications*.

[38] Craft N. E., Wise S. A., Soares J. H. (1993). Individual carotenoid content of SRM 1548 total diet and influence of storage temperature lyophilization, and irradiation on dietary carotenoids. *Journal of Agricultural and Food Chemistry*.

[39] Karppi J., Nurmi T., Olmedilla-Alonso B., Granado-Lorencio F., Nyyssönen K. (2008). Simultaneous measurement of retinol, alpha-tocopherol and six carotenoids in human plasma by using an isocratic reversed-phase HPLC method. *Journal of Chromatography B: Analytical Technologies in the Biomedical and Life Sciences*.

[40] Olmedilla B., Granado F., Gil-Martinez E., Blanco I., Rojas-Hidalgo E. (1997). Reference values for retinol, tocopherol, and main carotenoids in serum of control and insulin-dependent diabetic Spanish subjects. *Clinical Chemistry*.

[41] Thumham D. I., Smith E., Flora P. S. (1988). Concurrent liquid-chromatographic assay of retinol, alpha-tocopherol, beta-carotene, alpha-carotene, lycopene and beta-cryptoxanthin in plasma with tocopherol acetate as internal standard. *Clinical Chemistry*.

[42] Nierenberg D. W., Nann S. L. (1992). A method for determining concentrations of retinol, tocopherol, and five carotenoids in human plasma and tissue samples. *The American Journal of Clinical Nutrition*.

[43] Talwar D., Ha T. K. K., Cooney J., Brownlee C., St. JO'Reilly D. (1998). A routine method for the simultaneous measurement of retinol, *α*-tocopherol and five carotenoids in human plasma by reverse phase HPLC. *Clinica Chimica Acta*.

[44] Gueguen S., Herbeth B., Siest G., Leroy P. (2002). An isocratic liquid chromatographic method with diode-array detection for the simultaneous determination of *µ*-tocopherol, retinol, and five carotenoids in human serum. *Journal of Chromatographic Science*.

[45] Khachik F., Spangler C. J., Smith J. C., Canfield L. M. (1997). Identification, quantification, and relative concentrations of carotenoids and their metabolites in human milk and serum. *Analytical Chemistry*.

[46] Lyan B., Azaïs-Braesco V., Cardinault N. (2001). Simple method for clinical determination of 13 carotenoids in human plasma using an isocratic high-performance liquid chromatographic method. *Journal of Chromatography B: Biomedical Sciences and Applications*.

[47] Lee B.-L., New A.-L., Ong C.-N. (2003). Simultaneous determination of tocotrienols, tocopherols, retinol, and major carotenoids in human plasma. *Clinical Chemistry*.

[48] Emenhiser C., Sander L. C., Schwartz S. J. (1995). Capability of a polymeric C_30_ stationary phase to resolve *cis-trans* carotenoid isomers in reversed-phase liquid chromatography. *Journal of Chromatography A*.

[49] Ferruzzi M. G., Sander L. C., Rock C. L., Schwartz S. J. (1998). Carotenoid determination in biological microsamples using liquid chromatography with a coulometric electrochemical array detector. *Analytical Biochemistry*.

[50] Schweigert F. J., Steinhagen B., Raila J., Siemann A., Peet D., Buscher U. (2003). Concentrations of carotenoids, retinol and *α*-tocopherol in plasma and follicular fluid of women undergoing IVF. *Human Reproduction*.

[51] Epler K. S., Ziegler R. G., Craft N. E. (1993). Liquid chromatographic method for the determination of carotenoids, retinoids and tocopherols in human serum and in food. *Journal of Chromatography B: Biomedical Sciences and Applications*.

[52] Lee B. L., Ong C. N. (2009). Comprehensive high-performance liquid chromatographic method for the measurements of lipophilic antioxidants in human plasma. *Journal of Chromatography A*.

[53] Gleize B., Steib M., André M., Reboul E. (2012). Simple and fast HPLC method for simultaneous determination of retinol, tocopherols, coenzyme Q_10_ and carotenoids in complex samples. *Food Chemistry*.

[54] Kanďár R., Drábková P., Myslíková K., Hampl R. (2014). Determination of retinol and *α*-tocopherol in human seminal plasma using an HPLC with UV detection. *Andrologia*.

[55] Mitrowska K., Vincent U., von Holst C. (2012). Separation and quantification of 15 carotenoids by reversed phase high performance liquid chromatography coupled to diode array detection with isosbestic wavelength approach. *Journal of Chromatography A*.

[56] Chasse G. A., Mak M. L., Deretey E. (2001). An ab initio computational study on selected lycopene isomers. *Journal of Molecular Structure: THEOCHEM*.

[57] Pellegrini N., Riso P., Porrini M. (2000). Tomato consumption does not affect the total antioxidant capacity of plasma. *Nutrition*.

[58] Porrini M., Riso P., Testolin G. (1998). Absorption of lycopene from single or daily portions of raw and processed tomato. *British Journal of Nutrition*.

[59] Gärtner C., Stahl W., Sies H. (1997). Lycopene is more bioavailable from tomato paste than from fresh tomatoes. *The American Journal of Clinical Nutrition*.

